# The Impact of Body Composition on Outcomes in NSCLC Patients Treated with Immune Checkpoint Inhibitors: A Systematic Review

**DOI:** 10.3390/cancers17172765

**Published:** 2025-08-25

**Authors:** Carina Golban, Septimiu-Radu Susa, Norberth-Istvan Varga, Cristiana-Smaranda Ivan, Patricia Ortansa Schirta, Nicolae Călin Schirta, Alina Gabriela Negru, Sorin Saftescu, Serban Mircea Negru

**Affiliations:** 1 Doctoral School, Department of General Medicine, “Victor Babes” University of Medicine and Pharmacy, Eftimie Murgu Square No. 2, 300041 Timisoara, Romania; carina.golban@umft.ro (C.G.); septimiu.susa@umft.ro (S.-R.S.); 2 Department of General Medicine, “Victor Babeş” University of Medicine and Pharmacy, Eftimie Murgu Square No. 2, 300041 Timisoara, Romania; smaranda.ivan@student.umft.ro; 3 Department of Internal Medicine, University Hospital of Zurich, Raemistrasse 100, 8091 Zurich, Switzerland; patricia.schirta@usz.ch; 4 Department of Internal Medicine, Medzentrum Pfungen Aerztehaus, Riedäckerstrasse 5, 8422 Pfungen, Switzerland; nicolae.schirta@hin.ch; 5 Department of Cardiology, “Victor Babeș” University of Medicine and Pharmacy, Eftimie Murgu Square No. 2, 300041 Timișoara, Romania; alinanegru@umft.ro; 6 Department of Oncology, Faculty of Medicine, “Victor Babeş” University of Medicine and Pharmacy Timisoara, Eftimie Murgu Square No. 2, 300041 Timisoara, Romania; sorin.saftescu@umft.ro (S.S.); serban.negru@umft.ro (S.M.N.)

**Keywords:** non-small cell lung cancer, NSCLC, BMI, sarcopenia, cachexia, body composition, PD-L1, PD-1, progression-free survival, immune checkpoint

## Abstract

**Background/Objectives:** Immune checkpoint inhibitors targeting the PD-1/PD-L1 axis have become a standard in the treatment of all stages of non-small lung cancer. Beyond tumor-intrinsic biomarkers like PD-L1 expression, evidence points to the role of patient-related factors, such as body mass index, sarcopenia, and cachexia. These body composition parameters may reflect metabolic reserve or even immune competence and could help stratify outcomes in patients treated with PD-1 and PD-L1. This systematic review aims to evaluate the impact of body composition—specifically BMI, pretreatment weight loss, sarcopenia, and cachexia—on clinical outcomes such as progression-free and overall survival in NSCLC patients treated with immune checkpoint inhibitors. **Methods:** A systematic literature search was conducted across multiple databases including PubMed, Google Scholar, and Science Direct. We included full-text original research articles (1 January 2020–1 May 2025) reporting clinical outcomes of NSCLC patients treated with PD-1 or PD-L1 inhibitors, in relation to body composition factors (BMI, pretreatment weight loss, sarcopenia, cachexia). Eligible studies involved adults (>18 years) and included observational cohorts or controlled trials; animal or in vitro studies were excluded. Data extraction and risk of bias assessments were performed independently by two reviewers, with discrepancies being resolved through a third one. **Results:** From 12,358 records identified, 21 studies met the inclusion criteria. Most were retrospective cohorts assessing the impact of pre-treatment weight loss, cachexia, and sarcopenia on ICI outcomes in NSCLC. These factors consistently predicted poorer survival and response, while BMI alone showed limited prognostic value. Considerable heterogeneity in body composition definitions and outcome reporting was observed. **Conclusions:** Body composition—particularly weight loss, cachexia, and sarcopenia—significantly impacts survival and response in NSCLC patients treated with ICIs. These factors reflect immune–metabolic dysfunction that may impair treatment efficacy. BMI alone is insufficient; routine assessment of muscle mass and cachexia could improve risk stratification.

## Simple Summary

This systematic review examined how body composition affects outcomes in patients with non-small cell lung cancer (NSCLC) treated with immune checkpoint inhibitors (ICIs). Across 21 studies, factors such as pre-treatment weight loss, cancer cachexia, and sarcopenia consistently predicted poorer survival and lower response rates to ICIs. These body composition factors reflect underlying inflammation and immune dysfunction that may impair treatment effectiveness. In contrast, BMI alone was found to be an inadequate predictor of outcomes. The findings highlight the importance of using more precise measures—such as muscle mass, weight trajectory, and cachexia status—in clinical practice to better stratify patient risk and guide supportive interventions. Further research is needed to determine whether improving body composition can enhance the effectiveness of immunotherapy in NSCLC.

## 1. Introduction

Non-small cell lung cancer (NSCLC) accounts for approximately 85% of lung cancer diagnoses and remains one of the leading causes of cancer-related mortality worldwide, with over 1.8 million deaths annually [1,2]. Despite advances in surgical techniques, radiotherapy, and systemic therapies, including targeted therapies for epidermal growth factor receptor (EGFR) and anaplastic lymphoma kinase (ALK) driver mutations, the prognosis for many patients with NSCLC has remained poor, with 5-year survival rates often below 20% [3]. 

One of the main changes in approaching the treatment over the years is immunotherapy, specifically immune checkpoint inhibitors (ICIs) targeting the programmed death-1 (PD-1) receptor and its ligand PD-L1. They have reshaped the therapeutic landscape of NSCLC by restoring anti-tumor immune activity through the blockade of inhibitory pathways that suppress T-cell function [4,5]. PD-1 is an inhibitory receptor expressed on activated T cells, natural killer cells, and B cells. Upon binding to its ligand, PD-L1, a signal that attenuates immune cell activation is transmitted, thereby contributing to immune tolerance and tumor immune evasion [6]. ICIs such as PD-1 inhibitors (pembrolizumab, nivolumab) and PD-L1 inhibitors (atezolizumab, durvalumab) block this interaction, reactivating immune-mediated tumor clearance [7,8].

These agents showed significant survival benefits and are now approved for use either as monotherapy or combined with chemotherapy (chemoimmunotherapy) and as a first-line treatment for advanced NSCLC lacking EGFR or ALK driver mutations. Tumor PD-L1 expression guides eligibility for ICI monotherapy, but even among patients with PD-L1 (TPS) ≥ 50%, fewer than half respond [9,10]. Combining chemotherapy with ICIs can increase the overall response rate (ORR) to approximately 50%, yet only 20–25% of these patients achieve long-term responses. Consequently, the 2-year overall survival rate for NSCLC patients treated with ICIs is around 50% or lower [11,12]. A deeper understanding of predictive factors for response and prognosis is essential to identify patients most likely to benefit from ICIs and to develop new immunotherapy strategies to enhance outcomes for those with suboptimal responses. 

In this context, increasing attention has been directed to patient-specific characteristics such as body composition, as potential modulators of ICI effectiveness [13,14,15]. Parameters including body mass index (BMI), sarcopenia, pretreatment weight loss, and cachexia may reflect an individual’s inflammatory status, metabolic reserve, and immune function [16,17,18]. In NSCLC, up to 60% of the patients exhibit sarcopenia at diagnosis [19,20], while cachexia affects up to 50% of advanced-stage patients [21]. Both conditions have been associated with worse survival and treatment tolerance [22,23,24]. In contrast, obesity (BMI ≥ 30 kg/m^2^) has been linked to improved ICI outcomes, a phenomenon known as the “obesity paradox” [25]. Potential explanations include enhanced T-cell priming due to chronic low-grade inflammation or altered drug pharmacokinetics in obese individuals [26]. For instance, obese NSCLC patients with high PD-L1 expression treated with pembrolizumab have shown improved response rates and longer progression-free survival compared to their normal weight counterparts [27]. On the other side, sarcopenia and cachexia may impair immune responses by reducing cytokine production and T-cell activity, potentially diminishing ICI efficacy [28,29]. Interestingly, it has been speculated that sex-related differences in body composition may also influence immunotherapy outcomes [30]. For instance, studies in melanoma have shown that males with higher BMI appear to derive greater benefit from ICIs than females with comparable BMI, possibly due to differences in fat distribution, hormone signaling, and muscle mass composition [31,32].

This systematic review aims to evaluate the current clinical evidence regarding the association between body composition, specifically BMI, pretreatment weight loss sarcopenia and cachexia, and the clinical outcomes in NSCLC patients treated with PD-1 or PD-L1 inhibitors. By examining data across all disease stages and therapeutic settings, this review seeks to clarify the potential role of body composition as a prognostic and predictive factor in the immunotherapy era.

## 2. Materials and Methods

### 2.1. Guidelines and PICO

The present study followed the guidelines of the Preferred Reporting Items for Systematic Reviews and Meta-Analyses (PRISMA) 2020 statement [33]. The PRISMA checklist with the requested information is available in the Supplementary Materials (Table S1, PRISMA checklist). This review has not been prospectively registered in any database. We described the PICO elements (population, intervention/index, comparison, and outcome) as follows:

- Participants: patients with NSCLC, across all stages (I–IV), treated with immune checkpoint inhibitors.
- Intervention/Index: treatment with PD-1 or PD-L1 inhibitors (e.g., pembrolizumab, nivolumab, atezolizumab, durvalumab).
- Comparison: variations in body composition indicators (BMI, sarcopenia, cachexia).
- Outcome: clinical outcomes such as progression-free survival (PFS), overall survival (OS), and response rate (RR).

### 2.2. Search Strategy

A comprehensive literature search was conducted with the use of PubMed, Google Scholar, and Science Direct search engines. This multiplatform approach was chosen to overcome the limitations of individual databases, such as Boolean operator restrictions on Science Direct.

The search strategy combined Medical Subject Headings (MeSH) and free-text terms. Keywords included “non-small cell lung cancer”, “NSCLC”, “immune checkpoint inhibitors”, “PD-1”, “PD-L1”, “pembrolizumab”, “nivolumab”, “atezolizumab”, “durvalumab”, “BMI”, “body composition”, “sarcopenia”, “cachexia”, “obesity”, “treatment outcome”, “overall survival”, “progression-free survival”, and “prognostic factor”. Boolean operators (AND, OR, NOT) were applied to search queries in order to refine results, for example:

(“Non-Small Cell Lung Cancer” [MeSH] OR “NSCLC”) AND (“PD-1” OR “PD-L1” OR “Checkpoint Inhibitor”) AND (“Body Composition” OR “BMI” OR “Sarcopenia” OR “Cachexia”) AND (“Treatment Outcome” OR “Survival”).

### 2.3. Selection of Articles

To ensure the quality and reliability of the included sources, two authors independently assessed the publications. Any disagreements were resolved through discussion or, when necessary, by consulting a third author. For the screening process, two independent reviewers (C-S.I. and P.O.S.) evaluated all records for eligibility. The inter-rater reliability, measured by Cohen’s Kappa, was 0.82, indicating a high level of agreement. Discrepancies were addressed through consensus or, if unresolved, by involving a third reviewer (C.G.).

### 2.4. Inclusion Criteria

Articles were selected based on the following inclusion criteria:

(1) Studies with participants > 18 years old.
(2) Studies with abstracts relevant to the potential impact of body composition factors (e.g., BMI, sarcopenia, or cachexia) on treatment response or prognosis in non-small cell lung cancer (NSCLC) treated with PD-1 or PD-L1 immune checkpoint inhibitors.
(3) Studies that report clinical outcomes such as OS, PFS, DFS, response rate, or toxicity.
(4) Full-text original research articles published in English between 1 January 2020 and 1 May 2025.
(5) Studies involving human participants, specifically observational cohort studies or controlled trials.

Findings from animal or in vitro studies were considered only for context within the Introduction or Discussion.

Sarcopenia is most commonly defined by skeletal muscle index (SMI) at the third lumbar vertebra (L3), calculated as muscle area divided by height^2^ (cm^2^/m^2^) using a Hounsfield unit threshold of −29 to +150; widely used cut-offs are <52.4 cm^2^/m^2^ for men and <38.5 cm^2^/m^2^ for women [34].

Cancer cachexia was defined as a weight loss of >5% of the total body weight or a body mass index of 2% within 6 months before starting treatment [35].

### 2.5. Exclusion Criteria

The exclusion criteria were as follows:

(1) Studies not focused on NSCLC or not assessing the relationship between body composition and immunotherapy outcomes.
(2) Articles published in languages other than English, without an available translation.
(3) Publications not appearing in peer-reviewed journals.
(4) Studies lacking published/accessible full-text (abstract-only).
(5) Publications with unsuitable formats, such as letters, case reports, editorials, conference abstracts, systematic reviews, or meta-analyses.

After finalizing the list of eligible studies, two independent reviewers (C.-S.I. and A.G.N.) extracted key data using a standardized table. Extracted information included study identifier (first author and year), study design, cohort size, patient characteristics, analytic methods, treatment details, and reported outcomes. Any missing or unclear data were documented as limitations in the critical appraisal.

Studies were selected for each synthesis based on the availability of the relevant data. Due to heterogeneity in data reporting across studies, missing data were not imputed or otherwise statistically handled; analyses were conducted using the available data as reported in the original publications.

### 2.6. Quality Assessment of the Studies

Two reviewers (S.-R.S. and C.C.H.) independently conducted a quality assessment of the included articles using the National Institutes of Health (NIH) Study Quality Assessment Tools (available at www.nhlbi.nih.gov/health-topics/study-quality-assessment-tools; accessed on 1 May 2025). Any discrepancies between reviewers were resolved through discussion with a third reviewer (C.G.). The results of the quality assessment are summarized in Supplemental Material, Table S1.

## 3. Results

**Figure 1:**
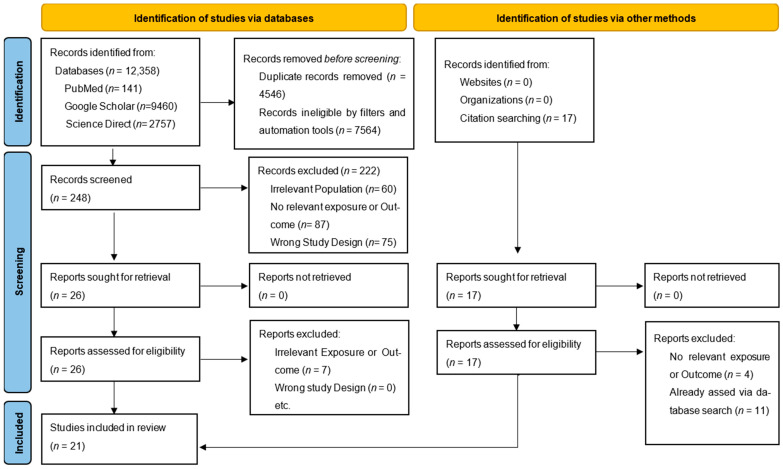
PRISMA flowchart of the study selection process.

The initial search, using keyword variants for body composition, sarcopenia, cachexia, BMI, and NSCLC with ICI therapy, identified 12,358 records across PubMed, ScienceDirect, and Google Scholar. ScienceDirect’s eight-operator limit required multiple queries, while PubMed and Google Scholar enabled refined searches. A 5-year timeframe (2020–2025) ensured relevance, reflecting data up to May 2025. Before screening, 7564 records were excluded using filters (e.g., language, study type) and automation tools targeting NSCLC and ICI studies. After deduplication, 4546 duplicates were removed, leaving 248 papers for abstract screening. Of these, 222 were excluded, and 26 underwent full-text review; out of these, 7 had irrelevant outcomes and 19 were deemed eligible. Snowballing citation searching added 2 additional studies, resulting in 21 studies included, as detailed in Figure 1.

**Figure 2:**
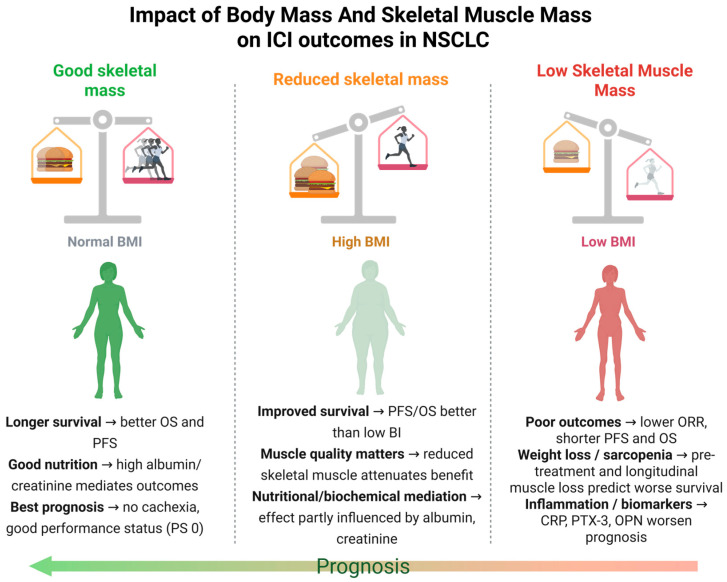
Impact of body mass and skeletal muscle mass on ICI outcomes in NSCLC.

The systematic review encompassed 21 studies, including a total of 5484 patients. Study populations varied, with patient numbers ranging from 47 [36] to 1791 [37], with male patients predominating (60–82% across studies). Most studies were retrospective (*n* = 16) [29,38,39,40,41,42,43,44,45,46,47,48,49,50,51,52], with prospective designs in four [36,53,54,55], and one that acquired data retrospectively and prospectively [37]. Body composition variables included sarcopenia (*n* = 13), cachexia (*n* = 8) or pretreatment weight loss (*n* = 3), and BMI (*n* = 7), assessed via CT-based muscle indices, weight loss thresholds (e.g., ≥5%), or BMI categories. Outcomes focused on progression-free survival (PFS) and overall survival (OS), with some including objective response rate (ORR). Sarcopenia and cachexia consistently predicted worse PFS and OS [41,47], while BMI findings varied, with underweight status linked to poorer outcomes [46] and obese BMI occasionally associated with better survival [46]. Studies spanned diverse regions (Japan, USA, Europe, China), with higher cachexia prevalence in patients with poor performance status or advanced disease [47,55]. Figure 2 summarizes the key relationships between body mass, skeletal muscle mass, and clinical outcomes in NSCLC patients treated with ICIs, highlighting the central findings of this systematic review. Moreover, Table 1 provides a detailed overview of the 21 studies (2020–2025) evaluating the impact of body composition variables, which have been included in this systematic review.

### 3.1. Impact of BMI on ICI Outcomes in NSCLC

Among the included studies, seven investigated the association between BMI and clinical outcomes in patients treated with PD-1/PD-L1 inhibitors, with varying findings [37,38,44,45,46,53,54] (Table 2). While findings vary, a recurring pattern suggests that both high and low BMI may influence clinical outcomes.

The studies by Antoun et al. [54], Jin et al. [45], and Lee et al. [46] highlighted that underweight status (BMI < 18.5 kg/m^2^) was significantly associated with worse survival outcomes. Specifically, Antoun et al. [54] reported that underweight patients had a significantly higher risk of mortality (HR = 1.65; 95% CI: 1.19–2.30) compared to those with normal weight, while Jin et al. [45] found that underweight male patients exhibited worse OS and PFS (HR = 5.35 for OS and 9.08 for PFS) compared to normal-weight males. Similarly, Lee et al. [46] noted that higher BMI, particularly in obese patients (BMI ≥ 25 kg/m^2^), was associated with a 25% lower risk of disease progression (HR = 0.75; 95% CI: 0.62–0.92), with a significant trend of decreasing progression risk with increasing BMI.

In contrast, some studies found no significant association between BMI and clinical outcomes. Minami et al. [38] reported that BMI and visceral adiposity indices, such as VSR and IMAC, were not predictive of ICI efficacy, although IMAC was a significant prognostic factor for OS (HR = 0.43; 95% CI: 0.18–0.998). Similarly, Chaunzwa et al. [37] found that baseline BMI was not significantly associated with OS or PFS in their large cohort of 1791 patients. Rounis et al. [53] reported a significant association between lower BMI and reduced ORR (*p* = 0.047), but this did not extend to PFS or OS. Liu et al. [44], however, found that obese BMI was significantly associated with longer PFS (*p* = 0.04), potentially mediated by nutritional status indicators such as serum albumin and creatinine. These findings suggest that while BMI may influence outcomes in certain contexts, its prognostic value varies across studies and patient populations, potentially due to differences in study design, sample size, or additional factors like nutritional status.

Collectively, these findings suggest that both extremes of BMI may have prognostic significance in patients treated with immune checkpoint inhibitors, but that assessments of muscle quality and nutritional status may provide additional prognostic value beyond BMI alone.

### 3.2. Prognostic Value of Pre-Treatment Weight Loss in NSCLC Immunotherapy

Three studies [43,45,54] consistently show that pre-treatment weight loss is a significant negative prognostic factor in patients treated with PD-1/PD-L1 inhibitors. Miyawaki et al. [43] reported that PWL was associated with a lower ORR (30% vs. 51%, *p* < 0.05), shorter PFS (2.3 vs. 12.0 months, *p* < 0.05), and shorter OS (10.8 vs. 23.9 months, *p* < 0.05) in 80 patients, with a 1.77-fold increased risk of progression and a 2.90-fold increased risk of death (95% CI: 1.01–3.10 and 1.40–6.00, respectively). Antoun et al. [54] found that PWL (≥11%) was linked to a higher risk of death (HR = 2.32; 95% CI: 1.61–3.35; *p* < 0.01) in 389 patients, with lesser degrees of weight loss showing a non-significant trend toward poorer outcomes. Jin et al. [45] confirmed worse PFS (*p* = 0.02) and OS (*p* = 0.0003) in 399 patients with PWL, with increased risks of progression (HR = 1.41; 95% CI: 1.05–1.90; *p* = 0.02) and death (HR = 1.99; 95% CI: 1.41–2.80; *p* < 0.0001), consistent across treatment lines and sexes, unaffected by adjustments for PD-L1 or creatinine.

These findings are summarized in Table 3.

These findings indicate that PWL is a predictor of poorer OS, PFS, and ORR in patients receiving immune checkpoint inhibitors, likely reflecting underlying factors such as cachexia or nutritional deficiency.

### 3.3. Cancer Cachexia and in NSCLC

Eight studies consistently showed that cancer cachexia is associated with poorer outcomes in NSCLC patients treated with ICIs [29,39,47,48,49,50,53,55]. Miyawaki et al. [39] reported that cachexia significantly reduced objective response rate (ORR = 15% vs. 57%, *p* < 0.001) and PFS (2.3 vs. 12.0 months, *p* < 0.001), with cachexia being an independent negative predictor of PFS. Similarly, Roch et al. [29] found that cachexia was associated with lower disease control (OR = 2.60, 95% CI: 1.03–6.58) and significantly shorter OS (HR = 6.26, 95% CI: 2.23–17.57). Rounis et al. [53] confirmed that cancer cachexia syndrome (CCS) independently predicted disease progression (OR = 8.11, 95% CI: 2.95–22.40) and poorer OS (HR = 2.52, 95% CI: 1.40–2.55). 

Madeddu et al. [55] further showed that IL-6 levels and miniCASCO-based cachexia severity were independent predictors of both PFS (HR = 1.04 and HR = 1.26) and OS (HR = 1.04 and HR = 2.38), supporting the prognostic role of inflammatory and nutritional alterations in cachexia. Matsuo et al. [47] demonstrated that cachexia was significantly associated with shorter PFS (2.1 vs. 5.1 months, *p* < 0.001) and OS (5.6 vs. 15.0 months, *p* < 0.001), with cachexia independently predicting OS (HR = 1.49, 95% CI: 1.02–2.18). 

In contrast, Murata et al. [48] observed shorter PFS and OS in cachectic patients, but these differences did not reach statistical significance. Tanimura et al. [49] reported that high-risk patients based on mGPS had markedly worse PFS (7.2 vs. 27.8 months, *p* = 0.001) and OS (19.6 months vs. not reached, *p* = 0.001). 

Finally, Kawachi et al. [50] showed that OS was significantly shorter in cachectic patients in both pembrolizumab monotherapy (17.2 vs. 35.8 months, *p* < 0.001) and ICI/chemotherapy groups (27.0 months vs. not reached, *p* = 0.044), highlighting the limited benefit of chemoimmunotherapy in cachectic patients with high PD-L1 expression. These findings consistently demonstrate the negative prognostic impact of cancer cachexia on response and survival in ICI-treated NSCLC patients (see Table 4).

### 3.4. Prognostic and Predictive Role of Sarcopenia in NSCLC Patients Treated with ICIs

Thirteen studies investigated the impact of sarcopenia, muscle mass, and muscle quality on clinical outcomes in NSCLC patients treated with ICIs [29,36,37,38,40,41,42,46,51,52,53,54,55]. Several studies demonstrated that low muscle mass or quality was significantly associated with poorer survival and response outcomes. For example, Roch et al. [29] reported that evolving sarcopenia was linked to a significantly shorter progression-free survival (PFS; HR 2.45, 95% CI: 1.09–5.53) and overall survival (OS; HR 3.87, 95% CI: 1.60–9.34). Similarly, Rounis et al. [53] found that baseline sarcopenia predicted significantly reduced PFS (2.96 vs. 7.96 months, *p* = 0.032) and OS (5.43 months vs. not reached, *p* = 0.006). Tenuta et al. [36] further showed that sarcopenic patients had a significantly higher risk of disease progression (OR 8.1, *p* = 0.011) and shorter PFS (20.3 vs. 61 weeks, *p* = 0.047).

Other studies highlighted the prognostic value of skeletal muscle quality over quantity. Minami et al. [38] showed that low IMAC, a marker of poor muscle quality, was an independent favorable prognostic factor for OS (HR 0.43, 95% CI: 0.18–0.998, *p* = 0.0496), while muscle quantity was not associated with PFS. Likewise, Nishioka et al. demonstrated that patients with high muscle quality had significantly higher ORR (35.0% vs. 15.8%, *p* < 0.05) and longer PFS (4.5 vs. 2.0 months, *p* < 0.05), without differences in OS or based on muscle quantity [40].

Dynamic changes in muscle mass during treatment were also shown to affect outcomes. Chaunzwa et al. [37] reported that a loss of skeletal muscle mass >5% was significantly associated with poorer OS and PFS (*p* = 0.03 and *p* = 0.001, respectively), while Kuno et al. [52] found that muscle maintenance was associated with significantly longer PFS during durvalumab treatment (29.2 vs. 11.3 months, *p* = 0.008), with muscle change confirmed as an independent predictor of superior PFS (HR 0.47, 95% CI: 0.25–0.90, *p* < 0.05). In contrast, several studies, such as Madeddu et al. [55], observed that although patients with progressive disease showed a greater decrease in skeletal muscle index (SMI, *p* = 0.003), SMI itself was not an independent predictor of progression (*p* = 0.515).

Additionally, sarcopenia was associated with immune-related adverse events (irAEs). Xue et al. [51] reported that sarcopenia significantly increased the risk of irAEs (OR 2.64, *p* = 0.03), and multivariate analysis confirmed sarcopenia as an independent predictor of irAEs (OR 5.67, *p* = 0.01), with the risk further elevated in sarcopenic patients with overweight/obesity. Overall, these findings, summarized in Table 5, consistently highlight that both baseline and evolving sarcopenia, as well as muscle quality and dynamic changes during treatment, are important prognostic and predictive factors in NSCLC patients undergoing ICI therapy.

Current evidence indicates that sarcopenia and poor skeletal muscle quality are consistent negative prognostic factors in NSCLC patients treated with ICIs. Baseline sarcopenia is associated with shorter PFS and OS, a higher risk of disease progression, and in some studies, lower response rates. Importantly, not only low muscle mass but also poor muscle quality and dynamic muscle loss during treatment have been shown to impact outcomes, suggesting that maintaining muscle mass and quality may support better ICI efficacy. Moreover, sarcopenia may increase the risk of immune-related adverse events (irAEs). These findings highlight the relevance of assessing body composition and monitoring muscle status during immunotherapy and suggest that nutritional and physical interventions to preserve muscle mass and function could represent a supportive strategy to improve clinical outcomes in this population.

## 4. Discussion

Body composition’s role in NSCLC immunotherapy efficacy has a complex role, one that this systematic approach tries to review. We found consistent evidence that cachexia, pre-treatment weight loss (PWL), and sarcopenia are associated with poorer outcomes—mainly, reduced PFS, OS, and ORR—in patients treated with ICIs, while BMI’s impact remains more heterogeneous and context-dependent. These findings align with the growing recognition that nutritional, metabolic, and inflammatory states fundamentally modulate immune competence and therapeutic response in cancer.

In interpreting the findings reported in Table 1, Table 2, Table 3 and Table 4, it is important to distinguish between statistically significant associations and those that are predictive at the individual patient level. Some associations, while statistically significant, may not reliably predict outcomes for a single patient, representing primarily scientific interest. In contrast, other associations demonstrate both statistical significance and predictive utility, typically quantified using hazard ratios with 95% confidence intervals. Highlighting this distinction shows the clinical relevance of the key findings and aids in translating them into patient-centered decision-making.

### 4.1. The Role of Body Composition in Immunotherapy Outcomes

Multiple studies demonstrated that pre-treatment weight loss (PWL), cancer cachexia, and low body mass index (BMI) are critical prognostic factors in NSCLC patients receiving immune checkpoint inhibitors (ICIs), reflecting systemic dysregulation that undermines antitumor immunity [29,39,43,45,47,48,49,50,53,54,55]. PWL and cachexia consistently predicted reduced response rates and shorter survival across diverse cohorts, with cachexia diminishing ICI benefit even in patients with high PD-L1 expression [50]. These facts suggest that host-related factors, driven by inflammatory cytokines such as IL-6 and TNF-α, impair T-cell function and ICI efficacy [55,56,57]. Similarly, underweight status (BMI < 18.5 kg/m^2^) was associated with worse outcomes, further indicating that nutritional deficits amplify the negative prognostic impact of cachexia and PWL [39,45,54]. The consistent results across sexes and treatment lines show the importance of routine assessment of these factors in clinical practice to optimize patient stratification. Whether PWL and cachexia specifically predict ICI resistance or simply reflect broader poor prognosis remains unresolved, partly due to heterogeneity in study designs and definitions. Interventions targeting cachexia—such as anti-IL-6 therapies (e.g., tocilizumab) or structured nutritional support—represent promising directions to enhance ICI outcomes and warrant further investigation in randomized trials [58]. Incorporating systematic screening for cachexia, PWL, and nutritional status into routine clinical workflows may help personalize immunotherapy strategies and identify patients who could benefit from supportive interventions.

### 4.2. Sarcopenia, Muscle Quality, and Treatment Response

Beyond cachexia and weight loss, sarcopenia—reflecting loss of skeletal muscle mass and quality—is starting to be considered as a key prognostic and potentially predictive factor in NSCLC patients treated with ICIs.

Our review shows that both baseline sarcopenia and dynamic muscle loss during treatment consistently predict poorer PFS and OS across multiple studies [29,36,37,41,42,46,51,52,53,54,55], sustained by prior evidence that skeletal muscle depletion reflects systemic inflammation, metabolic dysfunction, and immune suppression [59,60,61]. Importantly, the study by Minami et al. [38] highlights that muscle quality, as measured by IMAC, may offer superior prognostic value over muscle quantity alone, likely capturing the functional and immunometabolic competence of muscle tissue [60,62]. Dynamic muscle loss during treatment further correlated with poor survival, suggesting treatment-induced catabolism exacerbates systemic stress [37,63].

Beyond survival outcomes, sarcopenia may also impact toxicity risk. Xue et al. [49] showed that sarcopenic patients have a significantly higher incidence of immune-related adverse events (irAEs), potentially due to disrupted cytokine balance, impaired drug metabolism, and immune dysregulation [64]. While whether sarcopenia drives through ICI resistance remains uncertain, its independent prognostic and predictive impact strongly supports incorporating routine skeletal muscle assessment—both at baseline and dynamically during treatment—into immunotherapy workflows. Interventions such as resistance training, nutritional support, and anti-inflammatory agents could reduce sarcopenia’s negative effects, warranting further investigation to enhance ICI outcomes in NSCLC [65,66,67].

### 4.3. BMI: Obesity Paradox or Confounder?

The prognostic role of BMI in NSCLC patients treated with ICIs remains uncertain. Several studies [45,46,54] found that underweight status (BMI < 18.5 kg/m^2^) predicts poorer outcomes, likely reflecting nutritional deficits and systemic inflammation [68]. Lee et al. [46] and reported improved PFS in obese patients, consistent with the reported “obesity paradox” in ICI therapy [69,70]. However, other studies [35,36,51] found no consistent benefit of higher BMI after adjusting for muscle mass or inflammatory status.

These discrepancies highlight BMI’s limitations: it fails to differentiate fat from muscle mass or account for cachexia-related inflammation—factors shown to more directly impact immunotherapy outcomes [58,71]. Indeed, Chaunzwa et al. [37] demonstrated that dynamic muscle loss outperforms BMI in prognostic relevance. 

While BMI is a convenient and widely used metric, it does not necessarily reflect a patient’s physical fitness, muscle mass, or overall conditioning. Therefore, interpretation of weight changes should consider whether a reduction in BMI is recent or long-term, as this distinction may indicate whether the change is related to the underlying diagnosis, treatment, or other factors. Additionally, categorizing patients according to their baseline BMI—such as overweight, obese, or morbidly obese—provides important context, since the clinical significance of weight loss differs depending on the starting body mass. Reporting weight reduction in relative terms (percentage change) further enhances the relevance of these observations for prognosis, treatment planning, and risk stratification. Recent studies emphasize the limitations of BMI as a sole indicator of health, highlighting the importance of incorporating other measures, such as waist circumference and body composition analysis, to obtain a more comprehensive assessment of a patient’s health status [72,73].

Thus, while BMI remains a convenient marker, integrating more precise body composition and inflammatory metrics may better guide risk stratification in ICI-treated patients.

### 4.4. Clinical Implications and Future Directions

Our findings indicate that sarcopenia is a critical, yet underutilized, predictor of ICI outcomes in NSCLC, reflecting the patient’s immune–metabolic and inflammatory status. Additionally, we identified PWL and cachexia as significant predictors of ICI response. These parameters reflect the patient’s immune–metabolic and inflammatory status, which are key to effective anti-tumor responses [28,55]. In contrast, BMI alone provides limited prognostic value.

Routine pre-treatment assessment of muscle mass, muscle quality, and weight change should be integrated into clinical practice. Tools such as CT-based muscle indices and cachexia scores (e.g., mGPS, miniCASCO) can aid risk stratification and guide supportive interventions. Nutritional, exercise-based, and anti-inflammatory strategies to counteract cachexia and sarcopenia merit greater attention, with the potential to improve both immunotherapy outcomes and tolerability.

A future direction could be implementing targeted muscle physiotherapy before and after treatment. Prehabilitation may help maintain muscle mass and improve treatment tolerance, while post-treatment rehabilitation could support recovery, mitigate physical decline, and enhance quality of life.

Future prospective trials are needed to evaluate whether reversing cachexia can enhance ICI efficacy and to clarify the mechanisms linking muscle loss, inflammation, and immune dysfunction. Additionally, it would be of interest to assess whether these effects differ between patients receiving PD-1 inhibitors versus PD-L1 inhibitors, as the distinct mechanisms of action may interact differently with host factors such as systemic inflammation and muscle mass.

### 4.5. Limitations

Our systematic review has several limitations that should be acknowledged. The decision to include only studies from the past 5 years (2020–2025), while ensuring alignment with current clinical practice, may have excluded valuable older studies; however, this temporal focus also reduced noise from outdated findings. The majority of included studies were retrospective cohorts (17/21), introducing risks of selection bias, confounding, and heterogeneity in baseline characteristics. Definitions of body composition variables varied considerably—PWL thresholds, cachexia (weight loss, mGPS, or clinical diagnosis), and sarcopenia (diverse CT-based thresholds)—hindering direct comparisons and potentially contributing to outcome variability. Most studies relied on baseline body composition assessments; few evaluated dynamic changes, despite their emerging prognostic relevance. BMI analyses often lacked adjustment for inflammation or muscle mass, limiting insights into the “obesity paradox”. Although inflammatory markers such as IL-6 and CRP are mechanistically linked to cachexia and immune dysregulation, their inconsistent reporting across studies restricted meta-analytic synthesis. Similarly, adjustment for tumor burden, driver mutations, and comorbidities was limited, despite these factors’ known influence on ICI outcomes. 

In addition, this review focuses exclusively on NSCLC patients treated with immune checkpoint inhibitors. Consequently, the applicability of these findings to NSCLC patients who have not received ICIs remains uncertain. Future studies including such populations may provide further insights into the impact of body composition on treatment outcomes.

Finally, the geographic distribution was imbalanced, with a predominance of studies from Japan and Europe (*n* = 15), and fewer from the USA (*n* = 2), raising questions about global generalizability. These limitations highlight the need for future prospective, multicenter studies using standardized definitions, longitudinal body composition assessments, and more diverse populations to optimize the prognostic utility of PWL, cachexia, sarcopenia, and BMI in NSCLC patients treated with ICIs.

## 5. Conclusions

This systematic review demonstrates that body composition—particularly pre-treatment weight loss, cancer cachexia, and sarcopenia—has a significant prognostic impact on survival and response in NSCLC patients treated with ICIs. While pre-treatment weight loss and cancer cachexia also predict outcomes, these measures are often subjective or incomplete, whereas CT-based muscle mass provides an objective, real-time assessment, which is a better candidate for a future objective prognosis factor. This stands for the creation of objective, standardized, multifactorial prognostic scores. 

Sarcopenia reflects systemic inflammation and immune–metabolic dysfunction, which may undermine immunotherapy efficacy, even in patients with high PD-L1 expression. Conversely, BMI alone is an inadequate predictor, highlighting the need for precise body composition evaluation. Incorporating routine assessment of sarcopenia, along with weight trajectory and cachexia status, could improve risk stratification and inform supportive interventions. Prospective trials are warranted to determine whether stabilizing or improving body composition enhances ICI outcomes, and to further elucidate the biological mechanisms linking host factors and treatment response.

## Figures and Tables

**Table 1:** Overview table of the selected studies. NSCLC: Non-Small Cell Lung Cancer, ICI: Immune Checkpoint Inhibitor, PFS: Progression-Free Survival, OS: Overall Survival, ORR: Objective Response Rate, BMI: Body Mass Index, CCS: Cancer Cachexia Syndrome, SMI: Skeletal Muscle Index, VFI: Visceral Fat Index, SFI: Subcutaneous Fat Index, IMAC: Intramuscular Adipose Content, VSR: Visceral-to-Subcutaneous Ratio, PS: Performance Status, mGPS: Modified Glasgow Prognostic Score, SMa: Skeletal Muscle area, CRT: Chemoradiotherapy, CCRT: Concurrent Chemoradiotherapy, PTX-3: Pentraxin-3, OPN: Osteopontin, CRP: C-reactive Protein, PWL: Pretreatment Weight Loss.

Author	Year	Country	Study Design	Number of Patients ^1^	Number of Male Patients	ICI Agent(s)	Body Composition Variable	Outcomes Measured	Main Findings	Reference Number
Minami	2020	Japan	Retrospective cohort	74	48	PD-1/PD-L1 inhibitors	Sarcopenia, BMI, visceral adiposity	PFS, OS	Sarcopenia and IMAC are not predictive markers of ICI monotherapy NSCLC patients or PFS. IMAC was a prognostic factor for OS, but not PFS. VSR or sarcopenia is not associated with efficacy of ICI therapy.	[38]
Miyawaki	2020	Japan	Retrospective cohort	108	82	PD-1/PD-L1 inhibitors	Cachexia	PFS, OS, ORR	Patients with cachexia have a lower ORR, PFS, OS.	[39]
Nishioka	2020	Japan	Retrospective cohort	156	101	PD-1/PD-L1 inhibitors	Muscle quality	PFS, OS, ORR	Patients with decreased muscle quality had lower PFS and ORR.	[40]
Roch	2020	France	Retrospective cohort	142	93	PD-L1 inhibitors	Sarcopenia, Cachexia	PFS, OS	Cachexia–sarcopenia at beginning of therapy has a probability of shorter survival.	[29]
Takada	2020	Japan	Retrospective cohort	103	84	PD-1 inhibitors	SMI	PFS, OS	L3 muscle index Low ^2^ was an independent predictor of shorter PFS and OS.	[41]
Rounis	2021	Greece	Prospective cohort	83	70	PD-1/PD-L1 inhibitors	BMI, SMI, Cachexia	PFS, OS, ORR	Patients with lower BMI and CCS presence have lower ORR. CCS with lower OS and PFS.	[53]
Tenuta	2021	Italy	Prospective cohort	47	27	PD-1/PD-L1 inhibitors	Sarcopenia	PFS, OS	PFS and OS are shorter in sarcopenic patients in first-line ICI patients, with no difference in second-line.	[36]
Wang	2021	China	Retrospective cohort	105	73	PD-1 inhibitors	Sarcopenia	PFS, OS	Sarcopenia was significantly associated with poorer OS and PFS.	[42]
Miyawaki	2022	Japan	Retrospective cohort	80	61	PD-1/PD-L1 inhibitors	PWL	PFS, OS, ORR	Pre-treatment weight loss is associated with poorer OS, PFS, ORR. Weight loss is also an independent adverse prognostic factor for PFS and OS.	[43]
Antoun	2022	France	Prospective cohort	389	255	PD-1/PD-L1 inhibitors	BMI, PWL, SMI	OS	High BMI is linked to longer survival, disappearing when adjusted for confounders like tumor type, weight loss, and reduced skeletal muscle mass. Reduced skeletal muscle was linked to poor OS.	[54]
Liu	2022	China	Retrospective cohort	66	47	PD-1/PD-L1 inhibitors	BMI	PFS	Obese BMI associated with longer PFS. The effect of BMI on PFS may be mediated by serum albumin and creatinine levels, suggesting nutritional status influences immunotherapy outcomes.	[44]
Jin	2023	USA	Retrospective cohort	399	200	PD-1/PD-L1 inhibitors	BMI, PWL	PFS, OS	Underweight BMI and pretreatment weight loss predicted worse PFS and OS, especially in males and first-line ICI monotherapy; females had lower mortality risk.	[45]
Lee	2023	Korea	Retrospective cohort	820	630	PD-1/PD-L1 inhibitors	BMI, SMI, VFI	PFS, OS	Obese BMI is associated with improved OS and PFS. This association persisted after adjusting for SMI. No significant association was found between SMI, VFI, or SFI and OS/PFS after adjusting for BMI.	[46]
Madeddu	2023	Italy	Prospective cohort	74	54	PD-1 inhibitors	Cachexia, SMI	PFS, OS	Cachexia was an independent predictor of worse clinical response and shorter OS in NSCLC patients treated with ICIs. Patients with cachexia had significantly lower OS. Sarcopenia alone did not significantly affect survival outcomes.	[55]
Matsuo	2023	Japan	Retrospective cohort	183	135	PD-1/PD-L1 inhibitors	Cachexia	PFS, OS	Cachexia was linked to female sex, poor PS, never-smokers, and driver mutations, with poor PS and smoking. Cachexia patients had shorter PFS and OS. PS 0/without cachexia had the best PFS and OS. Cachexia worsened prognosis regardless of PD-L1, indicating its prognostic role in NSCLC with ICI therapy.	[47]
Murata	2023	Japan	Retrospective cohort	100	69	PD-1/PD-L1 inhibitors	Cachexia	PFS, OS	Cachexia was associated with worse PFS and OS, though differences were not statistically significant. Pre-treatment plasma analysis identified ghrelin, C-reactive protein, pentraxin-3, and osteopontin as factors associated with cachexia. Combinations of cachexia with high PTX-3 or OPN levels were predictive of worse PFS, while cachexia with high CRP or OPN levels were predictive of worse OS.	[48]
Tanimura	2023	Japan	Retrospective cohort	126	98	PD-L1 inhibitors	Cachexia	PFS, OS	Cachexia, assessed by mGPS, predicted worse outcomes in NSCLC patients on durvalumab post-CRT. High-risk patients had shorter PFS, OS, greater weight loss, and higher treatment discontinuation.	[49]
Chaunzwa	2024	USA	Retrospective and prospective cohort	1791	913	PD-1/PD-L1 inhibitors	BMI, Sarcopenia	PFS, OS	Loss of skeletal muscle mass (>5% reduction in SMa) during systemic therapy for advanced NSCLC was associated with worse OS and PFS, particularly in female patients across immunotherapy and chemoimmunotherapy cohorts. This muscle wasting, indicative of cancer cachexia, was a stronger prognostic marker than baseline body composition measures.	[37]
Kuno	2024	Japan	Retrospective cohort	98	73	PD-L1 inhibitors	Sarcopenia	PFS, OS	Longitudinal muscle loss (≥10% PMI reduction) during CCRT was associated with shorter PFS but not OS; single-point sarcopenia was not significantly predictive.	[52]
Xue	2024	China	Retrospective cohort	129	85	PD-1 inhibitors	Sarcopenia	PFS, OS	Sarcopenia was associated with significantly shorter OS and PFS.	[51]
Kawachi	2025	Japan	Retrospective cohort	411	320	PD-1/PD-L1 inhibitors	Cachexia	PFS, OS	Cancer cachexia was associated with shorter PFS and OS in the pembrolizumab group; no significant PFS difference in the ICI/chemotherapy group, but OS was shorter with cachexia. No significant differences in PFS or OS between pembrolizumab and ICI/chemotherapy after propensity score matching.	[50]

^1^ Treated with PD-1/PD-L1 inhibitors. ^2^ L3 muscle index. Low refers to low skeletal muscle mass at the L3 vertebra level.

**Table 2:** Impact of BMI on ICI outcomes in NSCLC patients across selected studies. PFS: progression-free survival, OS: overall survival, ORR: overall response rate, IMAC: Intramuscular adipose tissue content; VSR: Visceral-to-Subcutaneous fat ratio, BMI: Body mass index.

Author	Year	Country	Study Design	Number of Patients (Males)	ICI Agent(s)	Measured Outcomes	Main Findings	Reference Number
Minami	2020	Japan	Retrospective cohort	74 (48)	PD-1/PD-L1 inhibitors	PFS, OS	BMI and visceral adiposity indices (VSR, IMAC) were not predictive of ICI efficacy. BMI showed no significant association with OS. In contrast, IMAC was a significant prognostic factor for OS (HR = 0.43; 95% CI: 0.18–0.998; *p* = 0.0497).	[38]
Rounis	2021	Greece	Prospective cohort	83 (70)	PD-1/PD-L1 inhibitors	PFS, OS, ORR	Lower BMI was significantly associated with reduced clinical outcome ORR (*p* = 0.047), but not with PFS or OS.	[53]
Antoun	2022	France	Prospective cohort	389 (255)	PD-1/PD-L1 inhibitors	OS	Underweight status was significantly associated with worse survival compared to normal weight (HR = 1.65; 95% CI: 1.19–2.30), whereas overweight (HR = 0.94; 95% CI: 0.68–1.29) and obesity (HR = 0.69; 95% CI: 0.43–1.11) were not significantly associated with survival.	[54]
Liu	2022	China	Retrospective Cohort	66 (47)	PD-1/PD-L1 inhibitors	PFS	Obese BMI was significantly associated with longer (*p* = 0.04). The effect may be mediated by nutritional status indicators such as serum albumin and creatinine.	[44]
Jin	2023	USA	Retrospective Cohort	399 (200)	PD-1/PD-L1 inhibitors	PFS, OS	Progressive disease was significantly more common in underweight patients (BMI < 18.5 kg/m^2^; 67.7%) than in those with higher BMI (37.0%; *p* = 0.0008). Underweight male patients exhibited significantly worse outcomes, with an HR of 5.35 (95% CI: 2.42–11.81; *p* < 0.0001) for OS and 9.08 (95% CI: 4.19–16.69; *p* < 0.0001) for PFS compared to normal weight males.	[45]
Lee	2023	Korea	Retrospective Cohort	820 (630)	PD-1/PD-L1 inhibitors	PFS, OS	Patients with obesity (BMI ≥ 25 kg/m^2^) had a 25% lower risk of disease progression compared to those with normal BMI (Model 1: HR = 0.75; 95% CI: 0.62–0.92; *p* = 0.005). Furthermore, the risk of progression decreased significantly with increasing BMI, with each one standard deviation increase in BMI associated with a 12% reduction in the risk of progression (*p* = 0.001). The association observed with BMI remained significant after additional adjustments for SMI and VFI.	[46]
Chaunzwa	2024	USA	Retrospective and prospective cohort	1791 (913)	PD-1/PD-L1 inhibitors	PFS, OS	Baseline BMI was examined as a potential prognostic factor but was not significantly associated with OS or PFS.	[37]

**Table 3:** Pre-treatment weight loss impact in NSCLC patients treated with ICI. PFS: progression-free survival, OS: overall survival, ORR: overall response rate, PWL: Pre-Treatment Weight Loss.

Author	Year	Country	Study Design	Number of Patients (Males)	ICI Agent(s)	Measured Outcomes	Main Findings	Reference Number
Miyawaki	2022	Japan	Retrospective cohort	80 (61)	PD-1/PD-L1 inhibitors	PFS, OS, ORR	Patients with pre-treatment weight loss had a lower objective response rate (30% vs. 51%, *p* < 0.05), shorter PFS: 2.3 vs. 12.0 months, *p* < 0.05, and shorter OS: 10.8 vs. 23.9 months, *p* < 0.05, compared to those without weight loss. This weight loss was also associated with a 1.77-fold increased risk of disease progression and a 2.90-fold increased risk of death (95% CI: 1.01–3.10 and 1.40–6.00, respectively).	[43]
Antoun	2022	France	Prospective cohort	389 (255)	PD-1/PD-L1 inhibitors	OS	Baseline weight loss (≥11%) was associated with worse risk of death (HR = 2.32; 95% CI: 1.61–3.35; *p* < 0.01), while lower degrees of weight loss showed a non-significant trend toward poorer outcomes.	[54]
Jin	2023	USA	Retrospective Cohort	399 (200)	PD-1/PD-L1 inhibitors	PFS, OS	Both PFS and OS were significantly worse in patients with PWL compared to those without (*p* = 0.02 and *p* = 0.0003, respectively). PWL was also associated with increased risk of disease progression (HR: 1.41, 95% CI 1.05–1.90, *p* = 0.02) and death (HR: 1.99, 95% CI 1.41–2.80, *p* < 0.0001) compared to stable weight. Stratified analyses showed higher risk of progression with PWL in non-first-line monotherapy (HR: 1.73, *p* = 0.03), and higher risk of death in both first-line and non-first-line settings (HRs: 2.48 and 2.41). PWL also significantly increased risk of death in both sexes, with no association with progression. Adjusting for PD-L1 and creatinine did not alter results.	[45]

**Table 4:** The Prognostic Impact of Cancer Cachexia in NSCLC Patients Treated with ICIs. PFS: progression-free survival, OS: overall survival, ORR: objective response rate, PS: Performance Status, mGPS: Modified Glasgow Prognostic Score, CRP: C-reactive protein, OPN: Osteopontin, IL-6: interleukin-6, miniCASCO: mini-Cachexia Score, CCS: cancer cachexia syndrome, PTX-3: pentraxin-3, OPN: osteopontin, CRP: C-reactive protein, pre-CRT: before chemoradiotherapy.

Author	Year	Country	Study Design	Number of Patients (Males)	ICI Agent(s)	Measured Outcomes	Main Findings	Reference Number
Miyawaki	2020	Japan	Retrospective cohort	108 (82)	PD-1/PD-L1 inhibitors	PFS, OS, ORR	Patients with cachexia had a lower ORR (15% vs. 57%, *p* < 0.001) and shorter PFS (2.3 vs. 12.0 months, *p* < 0.001) compared to those without cachexia. Multivariate analysis confirmed cachexia as an independent negative predictor of PFS.	[39]
Roch	2020	France	Retrospective cohort	142 (93)	PD-L1 inhibitors	PFS, OS	Patients without cachexia had a higher probability of achieving disease control (59.9% vs. 41.1%; OR 2.60, 95% CI: 1.03–6.58), while patients with cachexia had significantly shorter OS (HR 6.26, 95% CI: 2.23–17.57).	[29]
Rounis	2021	Greece	Prospective cohort	83 (70)	PD-1/PD-L1 inhibitors	PFS, OS	The presence of cancer cachexia syndrome (CCS) was associated with lower response rates to ICIs (*p* ≤ 0.001) and was an independent predictor of disease progression (OR 8.11, 95% CI: 2.95–22.40, *p* ≤ 0.001). In multivariate analysis, baseline CCS independently predicted poorer survival (HR 2.52, 95% CI: 1.40–2.55, *p* = 0.002).	[53]
Madeddu	2023	Italy	Prospective cohort	74 (54)	PD-1 inhibitors	PFS, OS	For survival outcomes, IL-6 levels and miniCASCO-based cachexia severity were independent predictors of shorter PFS (HR 1.04, 95% CI: 1.02–1.05, *p* < 0.001 and HR 1.26, 95% CI: 1.09–1.46, *p* = 0.0024, respectively) and OS (HR 1.04, 95% CI: 1.02–1.06, *p* < 0.0001 and HR 2.38, 95% CI: 1.15–4.94, *p* = 0.0194, respectively). Kaplan–Meier analysis showed significantly lower OS in patients with cachexia (*p* = 0.0323) and higher cachexia severity (*p* = 0.0428). These findings support cachexia and its related inflammatory and nutritional alterations as key prognostic and predictive factors in patients treated with ICIs.	[55]
Matsuo	2023	Japan	Retrospective cohort	183 (135)	PD-1/PD-L1 inhibitors	PFS, OS	Cachexia was significantly associated with female sex (*p* = 0.011), poorer performance status (PS; *p* < 0.001), never-smoker status (*p* = 0.007), and driver mutations (*p* = 0.027), but not with histology, treatment line, PD-L1 expression, or PD-1/L1 inhibitor type. In multivariate analysis, poorer PS (OR 5.44, *p* < 0.001) and smoking status (OR 0.38, *p* = 0.012) remained independently associated with cachexia. Median PFS and OS were significantly shorter in cachectic patients (PFS: 2.1 vs. 5.1 months, *p* < 0.001; OS: 5.6 vs. 15.0 months, *p* < 0.001). Multivariate analysis identified cachexia as an independent predictor of poorer OS (HR 1.49, 95% CI: 1.02–2.18, *p* = 0.040) and PS and sex as significant predictors of PFS.	[47]
Murata	2023	Japan	Retrospective cohort	100 (69)	PD-1/PD-L1 inhibitors	PFS, OS	Cancer cachexia was significantly associated with sex, performance status, and smoking status (*p* = 0.001, 0.001, and 0.019, respectively). Median PFS (2.2 vs. 4.8 months, *p* = 0.247) and OS (7.1 vs. 13.1 months, *p* = 0.065) were shorter in cachectic patients, though differences were not statistically significant. Significant differences in PFS were observed with the combination of cachexia and PTX-3 (*p* = 0.008) or OPN (*p* = 0.030) expression, while combinations of cachexia with CRP (*p* = 0.038) or OPN (*p* < 0.001) levels were associated with significant differences in OS.	[48]
Tanimura	2023	Japan	Retrospective cohort	126 (98)	PD-L1 inhibitor	PFS, OS	High-risk patients had significantly worse PFS (7.2 vs. 27.8 months, *p* = 0.001) and OS (19.6 months vs. not reached, *p* = 0.001); pre-CRT mGPS was also predictive (PFS HR 1.45, OS HR 1.84).	[49]
Kawachi	2025	Japan	Retrospective cohort	411 (329)	PD-1/PD-L1 inhibitors	PFS, OS	OS was significantly shorter in cachectic patients vs. non-cachectic patients: 17.2 vs. 35.8 months (*p* < 0.001) in the pembrolizumab monotherapy group, and 27.0 months vs. not reached (*p* = 0.044) in the ICI/chemotherapy group; no OS difference between treatment groups after stratifying by cachexia status. ICI/chemotherapy offers limited benefit in patients with high PD-L1 expression and cancer cachexia.	[50]

**Table 5:** Impact of sarcopenia and muscle quality on ICI outcomes in NSCLC patients. PFS: progression-free survival, OS: overall survival, ORR: overall response rate, IMAC: Intramuscular Adipose Tissue Content, VSR: Visceral-to-Subcutaneous Fat Ratio, BMI: Body Mass Index, SMI: Skeletal Muscle Index, CT: Computed Tomography, irAEs: immune-related adverse events.

Author	Year	Country	Study Design	Number of Patients (Males)	ICI Agent(s)	Measured Outcomes	Main Findings	Reference Number
Minami	2020	Japan	Retrospective cohort	74 (48)	PD-1/PD-L1 inhibitors	PFS, OS, ORR	Skeletal muscle quantity and quality were not associated with PFS; however, low IMAC was an independent, favorable prognostic factor for OS (HR 0.43, 95% CI: 0.18–0.998, *p* = 0.0496), suggesting that muscle quality may be more relevant than quantity in predicting ICI outcomes.	[38]
Nishioka	2020	Japan	Retrospective cohort	156 (101)	PD-1/PD-L1 inhibitors	PFS, OS	Patients with high muscle quality had higher ORR (35.0% vs. 15.8%, *p* < 0.05) and longer PFS (4.5 vs. 2.0 months, *p* < 0.05), while muscle quantity was not associated with ORR or PFS. No differences in OS were observed based on muscle quality or quantity.	[40]
Roch	2020	France	Prospective cohort	142 (93)	PD-1/PD-L1 inhibitors	PFS, OS	Patients with evolving sarcopenia had significantly shorter PFS (HR 2.45, 95% CI: 1.09–5.53) and OS (HR 3.87, 95% CI: 1.60–9.34).	[29]
Takada	2020	Japan	Retrospective cohort	103 (84)	PD-1 inhibitors	OS	Low L3 muscle index was an independent predictor of shorter PFS (*p* = 0.0399) and OS (*p* = 0.0155). Disease control rate was significantly lower in the low vs. high L3 muscle index group (49.0% vs. 73.1%, *p* = 0.0117), while ORR did not differ significantly.	[41]
Rounis	2021	Greece	Prospective cohort	83 (70)	PD-1/PD-L1 inhibitors	PFS, OS	Baseline sarcopenia (low LSMI) was associated with significantly shorter PFS (2.96 vs. 7.96 months, *p* = 0.032) and OS (5.43 months vs. not reached, *p* = 0.006), while >5% LSMI reduction during ICI treatment had no impact on PFS or OS.	[53]
Tenuta	2021	Italy	Prospective cohort	47 (27)	PD-1/PD-L1 inhibitors	PFS, OS	Sarcopenic patients had significantly shorter PFS (20.3 vs. 61 weeks, *p* = 0.047) and an 8.1-fold higher risk of disease progression (OR 8.1, *p* = 0.011) compared to non-sarcopenic patients.	[36]
Wang	2021	China	Retrospective cohort	105 (73)	PD-1 inhibitors	OS	Sarcopenic patients receiving salvage anti-PD-1 therapy had significantly shorter PFS (2.67 vs. 7.96 months, *p* < 0.001) and OS (9.08 vs. 21.84 months, *p* < 0.001); sarcopenia was also associated with higher NLR (*p* = 0.041), which predicted OS.	[42]
Antoun	2022	France	Prospective cohort	389 (255)	PD-1/PD-L1 inhibitors	OS	Reduced skeletal muscle was associated with shorter survival in NSCLC (*p* = 0.02). In multivariate analysis, low SMI showed a non-significant trend toward worse OS (HR 1.16, 95% CI: 0.87–1.54), while high SMI showed a trend toward better OS (HR 0.73, 95% CI: 0.52–1.03, *p* = 0.07).	[54]
Lee	2023	Korea	Retrospective cohort	820 (630)	PD-1/PD-L1 inhibitors	PFS, OS	Low skeletal muscle index was associated with shorter OS in NSCLC (*p* = 0.02). In multivariate analysis adjusting for SMI, BMI remained a significant predictor of OS (HR 0.81, 95% CI: 0.73–0.91, *p* < 0.001) and PFS (HR 0.91, 95% CI: 0.82–0.99, *p* = 0.048); obesity was protective for OS (HR 0.68, *p* = 0.002) and borderline for PFS (HR 0.81, *p* = 0.064)	[46]
Madeddu	2023	Italy	Prospective cohort	74 (54)	PD-1/PD-L1 inhibitors	PFS, OS	Patients with PD showed a significantly greater decrease in SMI (*p* = 0.003); however, SMI was not an independent predictor of progression (OR 0.99, 95% CI: 0.95–1.03, *p* = 0.515).	[55]
Chaunzwa	2024	USA	Retrospective cohort	1791 (913)	PD-1/PD-L1 inhibitors	PFS, OS	Loss of skeletal muscle mass > 5% (ΔSMa >5%) was significantly associated with poorer OS and PFS (*p* = 0.03, respectively, *p* = 0.001 Kaplan–Meier analysis)	[37]
Kuno	2024	Japan	Retrospective cohort	98 (73)	PD-L1 inhibitors	PFS, OS	Patients with muscle maintenance had significantly longer PFS during durvalumab treatment (29.2 vs. 11.3 months, *p* = 0.008); multivariable analysis confirmed muscle change as an independent predictor of superior PFS (HR 0.47, 95% CI: 0.25–0.90, *p* < 0.05). No significant difference in OS was observed (*p* = 0.49).	[52]
Xue	2024	China	Retrospective cohort	129 (85)	PD-1 inhibitors	PFS, OS	Sarcopenia was associated with increased risk of irAEs (OR 2.64, *p* = 0.03); multivariate analysis confirmed sarcopenia as an independent predictor of irAEs (OR 5.67, *p* = 0.01).	[51]

## Data Availability

The data generated or analyzed during this study are included in this published article or are available from the corresponding author on reasonable request.

## Supplementary Materials

The following supporting information can be downloaded at: https://www.mdpi.com/article/10.3390/cancers17172765/s1, Supplemental Table S1: Quality Assessment of Included Studies.

## References

[1] World Health Organization (2025). WHO Cancer Fact Sheet.

[2] International Agency for Research on Cancer Global Cancer Observatory: Cancer Today. Lyon, France, Statistical Report, 2020. https://gco.iarc.fr/today.

[3] Araghi M., Mannani R., Maleki A.H., Hamidi A., Rostami S., Safa S.H., Faramarzi F., Khorasani S., Alimohammadi M., Tahmasebi S. (2023). Recent advances in non-small cell lung cancer targeted therapy; an update review. Cancer Cell Int..

[4] Zhang A., Fan T., Liu Y., Yu G., Li C., Jiang Z. (2024). Regulatory T cells in immune checkpoint blockade antitumor therapy. Mol. Cancer.

[5] Gang X., Yan J., Li X., Shi S., Xu L., Liu R., Cai L., Li H., Zhao M. (2024). Immune checkpoint inhibitors rechallenge in non-small cell lung cancer: Current evidence and future directions. Cancer Lett..

[6] Lin X., Kang K., Chen P., Zeng Z., Li G., Xiong W., Yi M., Xiang B. (2024). Regulatory mechanisms of PD-1/PD-L1 in cancers. Mol. Cancer.

[7] Han Y., Liu D., Li L. (2020). PD-1/PD-L1 pathway: Current researches in cancer. Am. J. Cancer Res..

[8] Jiang X., Wang J., Deng X., Xiong F., Ge J., Xiang B., Wu X., Ma J., Zhou M., Li X. (2019). Role of the tumor microenvironment in PD-L1/PD-1-mediated tumor immune escape. Mol. Cancer.

[9] Ricciuti B., Elkrief A., Alessi J.V.M., Wang X., Barrichello A.P.d.C., Pecci F., Lamberti G., Lindsay J., Sharma B., Felt K. (2022). Three-year outcomes and correlative analyses in patients with non–small cell lung cancer (NSCLC) and a very high PD-L1 tumor proportion score (TPS) ≥ 90% treated with first-line pembrolizumab. J. Clin. Oncol..

[10] Velcheti V., Rai P., Kao Y.-H., Chirovsky D., Nunes A.T., Liu S.V. (2024). 5-Year Real-World Outcomes with Frontline Pembrolizumab Monotherapy in PD-L1 Expression ≥ 50% Advanced NSCLC. Clin. Lung Cancer.

[11] Qin H., Yan H., Chen Y., Xu Q., Huang Z., Jiang W., Wang Z., Deng L., Zhang X., Zhang L. (2024). Clinical outcomes for immune checkpoint inhibitors plus chemotherapy in non-small-cell lung cancer patients with uncommon driver gene alterations. BMC Cancer.

[12] Reck M., Ciuleanu T.-E., Schenker M., Bordenave S., Cobo M., Juan-Vidal O., Reinmuth N., Richardet E., Felip E., Menezes J. (2024). Five-year outcomes with first-line nivolumab plus ipilimumab with 2 cycles of chemotherapy versus 4 cycles of chemotherapy alone in patients with metastatic non-small cell lung cancer in the randomized CheckMate 9LA trial. Eur. J. Cancer.

[13] Ma W., Shi Q., Zhang L., Qiu Z., Kuang T., Zhao K., Wang W. (2024). Impact of baseline body composition on prognostic outcomes in urological malignancies treated with immunotherapy: A pooled analysis of 10 retrospective studies. BMC Cancer.

[14] Kuang T., Zhang L., Qiu Z., Zhang Y., Wang W. (2023). Prognostic value of body composition on survival outcomes in melanoma patients receiving immunotherapy. Front. Immunol..

[15] Ishihara H., Nishimura K., Ikeda T., Fukuda H., Yoshida K., Iizuka J., Kondo T., Takagi T. (2024). Impact of body composition on outcomes of immune checkpoint inhibitor combination therapy in patients with previously untreated advanced renal cell carcinoma. Urol. Oncol. Semin. Orig. Investig..

[16] da Fonseca G.W.P., Farkas J., Dora E., von Haehling S., Lainscak M. (2020). Cancer Cachexia and Related Metabolic Dysfunction. Int. J. Mol. Sci..

[17] Müller M.J., Baracos V., Bosy-Westphal A., Dulloo A.G., Eckel J., Fearon K.C.H., Hall K.D., Pietrobelli A., Sørensen T.I.A., Speakman J. (2014). Functional body composition and related aspects in research on obesity and cachexia: Report on the 12th Stock Conference held on 6 and 7 September 2013 in Hamburg, Germany. Obes. Rev..

[18] Rey M., le Bacquer O., Mulliez A., Becaud J., Puechmaille M., Chanchou M., Mallet F., Mom T., Saroul N. (2025). Relationship between muscular mass, inflammatory status, tumor metabolic activity and oral intake in head and neck cancer at the outset of management. Clin. Nutr. ESPEN.

[19] Dong X., Dan X., Yawen A., Haibo X., Huan L., Mengqi T., Linglong C., Zhao R. (2020). Identifying sarcopenia in advanced non-small cell lung cancer patients using skeletal muscle CT radiomics and machine learning. Thorac. Cancer.

[20] Metelo-Liquito L.D., Solomon C., Bhana-Nathoo D. (2022). The prevalence of sarcopenia amongst non-small cell lung cancer patients, assessed using computed tomography, prior to treatment in a South African setting. S. Afr. J. Oncol..

[21] Hakozaki T., Nolin-Lapalme A., Kogawa M., Okuma Y., Nakamura S., Moreau-Amaru D., Tamura T., Hosomi Y., Takeyama H., Richard C. (2022). Cancer Cachexia among Patients with Advanced Non-Small-Cell Lung Cancer on Immunotherapy: An Observational Study with Exploratory Gut Microbiota Analysis. Cancers.

[22] Jensen S., Bloch Z., Quist M., Hansen T.T.D., Johansen C., Pappot H., Suetta C., Rafn B.S. (2023). Sarcopenia and loss of muscle mass in patients with lung cancer undergoing chemotherapy treatment: A systematic review and meta-analysis. Acta Oncol..

[23] Katsui K., Ogata T., Sugiyama S., Yoshio K., Kuroda M., Hiraki T., Kiura K., Maeda Y., Toyooka S., Kanazawa S. (2021). Sarcopenia is associated with poor prognosis after chemoradiotherapy in patients with stage III non-small-cell lung cancer: A retrospective analysis. Sci. Rep..

[24] Yue M., Qin Z., Hu L., Ji H. (2024). Understanding cachexia and its impact on lung cancer and beyond. Chin. Med J. - Pulm. Crit. Care Med..

[25] Zhang T., Li S., Chang J., Qin Y., Li C. (2023). Impact of BMI on the survival outcomes of non-small cell lung cancer patients treated with immune checkpoint inhibitors: A meta-analysis. BMC Cancer.

[26] Assumpção J.A.F., Pasquarelli-Do-Nascimento G., Duarte M.S.V., Bonamino M.H., Magalhães K.G. (2022). The ambiguous role of obesity in oncology by promoting cancer but boosting antitumor immunotherapy. J. Biomed. Sci..

[27] Cortellini A., Ricciuti B., Tiseo M., Bria E., Banna G.L., Aerts J.G., Barbieri F., Giusti R., Cortinovis D.L., Migliorino M.R. (2020). Baseline BMI and BMI variation during first line pembrolizumab in NSCLC patients with a PD-L1 expression ≥ 50%: A multicenter study with external validation. J. Immunother. Cancer.

[28] Wang J., Cao L., Xu S. (2020). Sarcopenia affects clinical efficacy of immune checkpoint inhibitors in non-small cell lung cancer patients: A systematic review and meta-analysis. Int. Immunopharmacol..

[29] Roch B., Coffy A., Jean-Baptiste S., Palaysi E., Daures J.-P., Pujol J.-L., Bommart S. (2020). Cachexia-sarcopenia as a determinant of disease control rate and survival in non-small lung cancer patients receiving immune-checkpoint inhibitors. Lung Cancer.

[30] Cohen S.F., Cruiziat D., Naimer J., Cohen V., Kasymjanova G., Spatz A., Agulnik J. (2024). Sex-Related Differences in Immunotherapy Outcomes of Patients with Advanced Non-Small Cell Lung Cancer. Curr. Oncol..

[31] McQuade J.L., Daniel C.R., Hess K.R., Mak C., Wang D.Y., Rai R.R., Park J.J., Haydu L.E., Spencer C., Wongchenko M. (2018). Association of body-mass index and outcomes in patients with metastatic melanoma treated with targeted therapy, immunotherapy, or chemotherapy: A retrospective, multicohort analysis. Lancet Oncol..

[32] Vick L.V., Rosario S., Riess J.W., Canter R.J., Mukherjee S., Monjazeb A.M., Murphy W.J. (2024). Potential roles of sex-linked differences in obesity and cancer immunotherapy: Revisiting the obesity paradox. npj Metab. Health Dis..

[33] Page M.J., McKenzie J.E., Bossuyt P.M., Boutron I., Hoffmann T.C., Mulrow C.D., Shamseer L., Tetzlaff J.M., Akl E.A., Brennan S.E. (2021). The PRISMA 2020 statement: An updated guideline for reporting systematic reviews. BMJ.

[34] Ishida Y., Maeda K., Yamanaka Y., Matsuyama R., Kato R., Yamaguchi M., Nonogaki T., Shimizu A., Ueshima J., Murotani K. (2020). Formula for the Cross-Sectional Area of the Muscles of the Third Lumbar Vertebra Level from the Twelfth Thoracic Vertebra Level Slice on Computed Tomography. Geriatrics.

[35] Fearon K., Strasser F., Anker S.D., Bosaeus I., Bruera E., Fainsinger R.L., Jatoi A., Loprinzi C., MacDonald N., Mantovani G. (2011). Definition and classification of cancer cachexia: An international consensus. Lancet Oncol..

[36] Tenuta M., Gelibter A., Pandozzi C., Sirgiovanni G., Campolo F., Venneri M.A., Caponnetto S., Cortesi E., Marchetti P., Isidori A.M. (2021). Impact of Sarcopenia and Inflammation on Patients with Advanced Non-Small Cell Lung Cancer (NCSCL) Treated with Immune Checkpoint Inhibitors (ICIs): A Prospective Study. Cancers.

[37] Chaunzwa T.L., Qian J.M., Li Q., Ricciuti B., Nuernberg L., Johnson J.W., Weiss J., Zhang Z., MacKay J., Kagiampakis I. (2024). Body Composition in Advanced Non-Small Cell Lung Cancer Treated with Immunotherapy. JAMA Oncol..

[38] Minami S., Ihara S., Tanaka T., Komuta K. (2020). Sarcopenia and Visceral Adiposity Did Not Affect Efficacy of Immune-Checkpoint Inhibitor Monotherapy for Pretreated Patients with Advanced Non-Small Cell Lung Cancer. World J. Oncol..

[39] Miyawaki T., Naito T., Kodama A., Nishioka N., Miyawaki E., Mamesaya N., Kawamura T., Kobayashi H., Omori S., Wakuda K. (2020). Desensitizing Effect of Cancer Cachexia on Immune Checkpoint Inhibitors in Patients with Advanced NSCLC. JTO Clin. Res. Rep..

[40] Nishioka N., Naito T., Notsu A., Mori K., Kodama H., Miyawaki E., Miyawaki T., Mamesaya N., Kobayashi H., Omori S. (2021). Unfavorable impact of decreased muscle quality on the efficacy of immunotherapy for advanced non-small cell lung cancer. Cancer Med..

[41] Takada K., Yoneshima Y., Tanaka K., Okamoto I., Shimokawa M., Wakasu S., Takamori S., Toyokawa G., Oba T., Osoegawa A. (2021). Clinical impact of skeletal muscle area in patients with non-small cell lung cancer treated with anti-PD-1 inhibitors. J. Cancer Res. Clin. Oncol..

[42] Wang Y., Chen P., Huang J., Liu M., Peng D., Li Z., Chen T., Hong S., Zhou Y. (2021). Assessment of sarcopenia as a predictor of poor overall survival for advanced non-small-cell lung cancer patients receiving salvage anti-PD-1 immunotherapy. Ann. Transl. Med..

[43] Miyawaki T., Naito T., Yabe M., Kodama H., Nishioka N., Miyawaki E., Mamesaya N., Kobayashi H., Omori S., Wakuda K. (2022). Impact of weight loss on treatment with PD-1/PD-L1 inhibitors plus chemotherapy in advanced non-small-cell lung cancer. Support. Care Cancer.

[44] Liu Z., Diao Y., Li X. (2022). Body mass index and serum markers associated with progression-free survival in lung cancer patients treated with immune checkpoint inhibitors. BMC Cancer.

[45] Jin J., Visina J., Burns T.F., Diergaarde B., Stabile L.P. (2023). Male sex and pretreatment weight loss are associated with poor outcome in patients with advanced non-small cell lung cancer treated with immunotherapy: A retrospective study. Sci. Rep..

[46] Lee J.H., Kang D., Ahn J.S., Guallar E., Cho J., Lee H.Y. (2023). Obesity paradox in patients with non-small cell lung cancer undergoing immune checkpoint inhibitor therapy. J. Cachex- Sarcopenia Muscle.

[47] Matsuo N., Azuma K., Murotani K., Murata D., Matama G., Kawahara A., Kojima T., Tokito T., Hoshino T. (2023). Prognostic effect of cachexia in patients with non-small cell lung cancer receiving immune checkpoint inhibitors. Thorac. Cancer.

[48] Murata D., Azuma K., Matsuo N., Murotani K., Matama G., Kawahara A., Sasada T., Tokito T., Hoshino T. (2023). Survival and biomarkers for cachexia in non-small cell lung cancer receiving immune checkpoint inhibitors. Cancer Med..

[49] Tanimura K., Takeda T., Yoshimura A., Honda R., Goda S., Shiotsu S., Fukui M., Chihara Y., Uryu K., Takei S. (2023). Predictive Value of Modified Glasgow Prognostic Score and Persistent Inflammation among Patients with Non-Small Cell Lung Cancer Treated with Durvalumab Consolidation after Chemoradiotherapy: A Multicenter Retrospective Study. Cancers.

[50] Kawachi H., Yamada T., Tamiya M., Negi Y., Kijima T., Goto Y., Nakao A., Shiotsu S., Tanimura K., Takeda T. (2025). Clinical impact of cancer cachexia on the outcome of patients with non-small cell lung cancer with PD-L1 tumor proportion scores of ≥50% receiving pembrolizumab monotherapy versus immune checkpoint inhibitor with chemotherapy. OncoImmunology.

[51] Xue D., Li N., Yang J., Men K., Li L., Jiang H., Zhao X., Zhang S., Chen X. (2024). Sarcopenia predicts immune-related adverse events due to anti-PD-1/PD-L1 therapy in patients with advanced lung cancer. Front. Oncol..

[52] Kuno H., Nishioka N., Yamada T., Kunimatsu Y., Yoshimura A., Hirai S., Futamura S., Masui T., Egami M., Chihara Y. (2024). The Significance of Longitudinal Psoas Muscle Loss in Predicting the Maintenance Efficacy of Durvalumab Treatment Following Concurrent Chemoradiotherapy in Patients with Non-Small Cell Lung Cancer: A Retrospective Study. Cancers.

[53] Rounis K., Makrakis D., Tsigkas A.-P., Georgiou A., Galanakis N., Papadaki C., Monastirioti A., Vamvakas L., Kalbakis K., Vardakis N. (2021). Cancer cachexia syndrome and clinical outcome in patients with metastatic non-small cell lung cancer treated with PD-1/PD-L1 inhibitors: Results from a prospective, observational study. Transl. Lung Cancer Res..

[54] Antoun S., Lanoy E., Ammari S., Farhane S., Martin L., Robert C., Planchard D., Routier E., Voisin A.L., Messayke S. (2023). Protective effect of obesity on survival in cancers treated with immunotherapy vanishes when controlling for type of cancer, weight loss and reduced skeletal muscle. Eur. J. Cancer.

[55] Madeddu C., Busquets S., Donisi C., Lai E., Pretta A., López-Soriano F.J., Argilés J.M., Scartozzi M., Macciò A. (2023). Effect of Cancer-Related Cachexia and Associated Changes in Nutritional Status, Inflammatory Status, and Muscle Mass on Immunotherapy Efficacy and Survival in Patients with Advanced Non-Small Cell Lung Cancer. Cancers.

[56] Chen A.Y., Wolchok J.D., Bass A.R. (2021). TNF in the era of immune checkpoint inhibitors: Friend or foe?. Nat. Rev. Rheumatol..

[57] Huseni M.A., Wang L., Klementowicz J.E., Yuen K., Breart B., Orr C., Liu L.-F., Li Y., Gupta V., Li C. (2023). CD8+ T cell-intrinsic IL-6 signaling promotes resistance to anti-PD-L1 immunotherapy. Cell Rep. Med..

[58] Argilés J.M., López-Soriano F.J., Stemmler B., Busquets S. (2019). Therapeutic strategies against cancer cachexia. Eur. J. Transl. Myol..

[59] Ma S., Zhao G., Sui S., Chen X., Wu L., Wang T., Xu W., Lu Z., Wang A., Wu X. (2025). Tumor microenvironment and immune-related myositis: Addressing muscle wasting in cancer immunotherapy. Front. Immunol..

[60] VanderVeen B.N., Murphy E.A., Carson J.A. (2020). The Impact of Immune Cells on the Skeletal Muscle Microenvironment During Cancer Cachexia. Front. Physiol..

[61] Ferrer M., Anthony T.G., Ayres J.S., Biffi G., Brown J.C., Caan B.J., Feliciano E.M.C., Coll A.P., Dunne R.F., Goncalves M.D. (2023). Cachexia: A systemic consequence of progressive, unresolved disease. Cell.

[62] Bye A., Sjøblom B., Wentzel-Larsen T., Grønberg B.H., Baracos V.E., Hjermstad M.J., Aass N., Bremnes R.M., Fløtten Ø., Jordhøy M. (2017). Muscle mass and association to quality of life in non-small cell lung cancer patients. J. Cachex-Sarcopenia Muscle.

[63] Cole C.L., Kleckner I.R., Jatoi A., Schwarz E.M., Dunne R.F. (2018). The Role of Systemic Inflammation in Cancer-Associated Muscle Wasting and Rationale for Exercise as a Therapeutic Intervention. JCSM Clin. Rep..

[64] Nakamura R., Inage Y., Tobita R., Yoneyama S., Numata T., Ota K., Yanai H., Endo T., Inadome Y., Sakashita S. (2018). Sarcopenia in Resected NSCLC: Effect on Postoperative Outcomes. J. Thorac. Oncol..

[65] Zhang N., Zhai L., Wong R.M.Y., Cui C., Law S.-W., Chow S.K.-H., Goodman S.B., Cheung W.-H. (2024). Harnessing immunomodulation to combat sarcopenia: Current insights and possible approaches. Immun. Ageing.

[66] Zanetti M., Cappellari G.G., Barazzoni R., Sanson G. (2020). The Impact of Protein Supplementation Targeted at Improving Muscle Mass on Strength in Cancer Patients: A Scoping Review. Nutrients.

[67] Deng H.-Y., Chen Z.-J., Qiu X.-M., Zhu D.-X., Tang X.-J., Zhou Q. (2021). Sarcopenia and prognosis of advanced cancer patients receiving immune checkpoint inhibitors: A comprehensive systematic review and meta-analysis. Nutrition.

[68] Shepshelovich D., Xu W., Lu L., Fares A., Yang P., Christiani D., Zhang J., Shiraishi K., Ryan B.M., Chen C. (2019). Body Mass Index (BMI), BMI Change, and Overall Survival in Patients with SCLC and NSCLC: A Pooled Analysis of the International Lung Cancer Consortium. J. Thorac. Oncol..

[69] Nitsche L.J., Mukherjee S., Cheruvu K., Krabak C., Rachala R., Ratnakaram K., Sharma P., Singh M., Yendamuri S. (2022). Exploring the Impact of the Obesity Paradox on Lung Cancer and Other Malignancies. Cancers.

[70] Vedire Y., Kalvapudi S., Yendamuri S. (2023). Obesity and lung cancer—A narrative review. J. Thorac. Dis..

[71] Rupert J.E., Narasimhan A., Jengelley D.H., Jiang Y., Liu J., Au E., Silverman L.M., Sandusky G., Bonetto A., Cao S. (2021). Tumor-derived IL-6 and trans-signaling among tumor, fat, and muscle mediate pancreatic cancer cachexia. J. Exp. Med..

[72] Shanks C.B., Bruening M., Yaroch A.L. (2025). BMI or not to BMI? debating the value of body mass index as a measure of health in adults. Int. J. Behav. Nutr. Phys. Act..

[73] Palumbo A.M., Jacob C.M., Khademioore S., Sakib M.N., Yoshida-Montezuma Y., Christodoulakis N., Yassa P., Vanama M.S., Gamra S., Ho P. (2025). Validity of non-traditional measures of obesity compared to total body fat across the life course: A systematic review and meta-analysis. Obes. Rev..

