# Supplementary material for: The Impact of Body Composition on Outcomes in NSCLC Patients Treated with Immune Checkpoint Inhibitors: A Systematic Review
Source: Cancers (Basel). 2025 Aug 25;17(17):2765. doi: 10.3390/cancers17172765 (PMC12427566; doi:10.3390/cancers17172765)
Supplement: Supplementary file 1 [file cancers-17-02765-s001.zip › cancers-3771122-supplementary.pdf]

cancers

MDPI

Systematic Review

The Impact of Body Composition on Outcomes in NSCLC Patients Treated with Immune Checkpoint Inhibitors: A Systematic Review

Supplemental Table S1. Quality Assessment of Included Studies. Y = yes; N = no; CD = can not determine; NA = not applicable; NR = not reported; ROC=Retrospective Cohort Study; PCS=Prospective Cohort Study.

	Study Design	1	2	3	4	5	6	7	8	9	10	11	12	13	14	Quality
Minami (2020)[36]	ROC	Y	Y	CD	Y	N	Y	Y	Y	Y	N	Y	N	Y	Y	Good
Miyawaki (2020)[37]	ROC	Y	Y	CD	Y	N	Y	Y	Y	Y	N	Y	NR	Y	Y	Good
Nishioka (2020)[38]	ROC	Y	Y	Y	Y	N	Y	Y	Y	Y	N	Y	NR	Y	Y	Good
Roch (2020)[29]	ROC	Y	N	CD	Y	N	Y	Y	Y	Y	Y	Y	NR	Y	Y	Good
Takada (2020)[39]	ROC	Y	Y	Y	Y	N	Y	Y	Y	Y	Y	Y	NR	Y	Y	Good
Rounis (2021)[51]	PCS	Y	Y	Y	Y	N	Y	Y	Y	Y	Y	Y	NR	Y	Y	Good
Tenuta (2021)[34]	PCS	Y	Y	Y	Y	N	Y	Y	Y	Y	Y	Y	NR	Y	Y	Good
Wang (2021)[40]	ROC	Y	Y	Y	Y	N	Y	Y	Y	Y	Y	Y	NR	Y	Y	Good
Miyawaki (2022)[41]	ROC	Y	Y	Y	Y	N	Y	Y	Y	Y	N	Y	NR	Y	Y	Good
Antoun (2022)[52]	PCS	Y	Y	CD	Y	N	Y	Y	Y	Y	N	Y	Y	CD	Y	Good
Liu (2022)[42]	ROC	Y	Y	Y	Y	N	Y	Y	Y	Y	N	Y	NR	CD	Y	Good
Jin (2023)[43]	ROC	Y	Y	CD	Y	N	Y	Y	Y	Y	N	Y	NR	NR	Y	Good
Lee (2023)[44]	ROC	Y	Y	Y	Y	N	Y	Y	Y	Y	N	NR	Y	Y	Y	Good
Madeddu (2023)[53]	PCS	Y	Y	Y	Y	N	Y	Y	Y	Y	Y	Y	CD	Y	Y	Good
Matsuo (2023)[45]	ROC	Y	Y	CD	Y	N	Y	Y	Y	Y	N	Y	N	Y	Y	Good
Murata (2023)[46]	ROC	Y	Y	Y	Y	N	Y	Y	Y	Y	N	Y	NR	Y	Y	Good
Tanimura (2023)[47]	Y	Y	Y	Y	Y	N	Y	Y	Y	Y	N	Y	NR	Y	Y	Good
Chaunzwa (2024)[35]	Mixed	Y	Y	Y	Y	N	Y	Y	Y	Y	Y	Y	NR	Y	Y	Good

 https://doi.org/10.3390/cancers17172765

Kuno (2024)[50]	ROC	Y	Y	Y	Y	N	Y	Y	Y	Y	Y	Y	NR	Y	Y	Good
Xue (2024)[49]	ROC	Y	Y	Y	Y	N	Y	Y	Y	Y	Y	Y	NR	Y	Y	Good
Kawachi (2025)[48]	ROC	Y	Y	Y	Y	N	Y	Y	Y	Y	Y	Y	NR	CD	Y	Good

Disclaimer/Publisher's Note: The statements, opinions and data contained in all publications are solely those of the individual author(s) and contributor(s) and not of MDPI and/or the editor(s). MDPI and/or the editor(s) disclaim responsibility for any injury to people or property resulting from any ideas, methods, instructions or products referred to in the content.
